# Cost analysis of corneal tissue processing: A scoping review protocol

**DOI:** 10.1371/journal.pone.0317681

**Published:** 2025-02-10

**Authors:** Gustavo Moura Maidana, Marcos Antônio Ferreira Júnior, Felipe Machado Mota, Andréia Insabralde de Queiroz Cardoso, Bruna Dias Abes, Mayra Dias, Letícia Lima Meza

**Affiliations:** Universidade Federal de Mato Grosso do Sul, Campo Grande, Mato Grosso do Sul, Brasil; Harvard Medical School, UNITED STATES OF AMERICA

## Abstract

**Introduction:**

Diseases affecting the cornea are a group of pathological conditions responsible for the main causes of blindness worldwide. Corneal transplantation aims to replace dysfunctional corneal tissue with a transparent tissue graft obtained from a deceased donor, which enables the full recovery of lost vision. Processes are initially performed to prepare the corneal tissue for transplantation in order for this transplant to be viable. There is a gap in knowledge regarding the costs of these processes. This review aims to carry out a robust, broad and current mapping of studies which analyze the costs of processing corneal tissue for transplantation.

**Objective:**

The objective of this study is to map the evidence produced in the literature on cost analysis studies of corneal tissue processing.

**Materials and methods:**

A scoping review will be conducted to map the topic, gather different research designs and identify the available scientific evidence on corneal tissue processing. To this end, a scoping review protocol was developed, registered in the Open Science Framework (accessed at: DOI 10.17605/OSF.IO/2X89U), following the good practices described by the Joanna Briggs Institute. The review report will be guided by the Preferred Reporting Items for Systematic reviews and Meta-Analyses extension for Scoping Reviews checklist. The data will be presented descriptively, with a summary of the studies found. The guiding research question of the study is: What is the scope of scientific evidence on the cost analysis of corneal tissue processing for transplants?

## Introduction

Eye diseases are very common, with around 2.2 billion people suffering from some degree of visual impairment or blindness [1]. Among these, those affecting the cornea constitute a group of pathological conditions responsible for the main causes of blindness in the world [2]. The cornea is an avascular and transparent tissue, an important component of the refractive power of light for the retina. The only possible treatment for these conditions in certain cases is transplantation or keratoplasty, with replacement of the dysfunctional tissue with a graft from a deceased donor [2].

Keratoplasties involve individuals of working age, with an average age of 33.5 to 48.4 years among recipients, which exacerbates the economic impact of visual impairment, estimated at US$134.2 billion in the United States alone [3–5]. In addition, unequal distribution of appropriate treatments intensifies the difficulties faced by low-and middle-income countries, where resource scarcity represents a significant obstacle to addressing this problem [1].

Given the waiting lists for transplants and the scarcity of resources worldwide, understanding the costs involved in preparing tissues for transplantation is essential [6]. Each stage of this process requires efficient allocation of resources, and detailed understanding of the costs described in the global literature can provide essential support for more effective management policies and strategic adjustments in providing treatment.

A search was conducted on January 21, 2024, on the Cochrane Library, JBI Evidence Synthesis, Open Science Framework (OSF) and International Prospective Register of Ongoing Systematic Reviews (PROSPERO) platforms using the controlled Medical Subject Headings (MeSH) descriptors: Corneal Transplantation, Eye Bank, Eye Banks, Costs and Cost Analysis with the use of the Boolean operators “AND” and “OR”, but without results that covered the intersection of cost analysis and cornea processing for transplantation.

Therefore, it is clear that the topic requires further research to obtain results related to the cost of corneal tissue processing for transplantation in eye banks. This scoping review protocol aims to establish a clear method for conducting a transparent, consistent and reproducible study, with methods and procedures defined in advance to minimize potential biases. These principles will guide a review aimed at mapping the existing scientific evidence on the costs associated with corneal tissue processing for transplantation.

## Justification

A systematic review provided evidence on the costs associated with obtaining organs for transplantation, but did not identify studies on the costs involved in acquiring tissues for this purpose, such as corneas [7]. This highlighted a significant knowledge gap that could be addressed by a scoping review, as this search method aims to map both indexed and grey literature to provide an overview of a specific topic [8].

Since visual impairment and blindness are associated with reduced earnings, limited access to education, fewer employment opportunities and an increased risk of mortality [9], the need to investigate and analyze the costs associated with procedures to make corneas available for transplantation is highlighted.

## Materials and methods

A review will be conducted following the JBI Evidence Synthesis Handbook for Scoping Reviews, with the aim of mapping the topic [10]. To this end, this scoping review protocol was developed and registered with the OSF (accessed at: DOI 10.17605/OSF.IO/2X89U), in accordance with the good practice guidelines described by the Joanna Briggs Institute (JBI) [11]. The review report will be guided by the Preferred Reporting Items for Systematic reviews and Meta-Analyses extension for Scoping Reviews (PRISMA-ScR) checklist [12] (S1 and S3 Appendix).

The heterogeneity of the studies will be addressed by categorizing the different cost analysis methods used. Moreover, details about the currency used and the cost items of each study will be described and analyzed to make this information clear. The research question was developed using the Population, Concept, and Context (PCC) mnemonic [10], resulting in the following question:

What is the scope of scientific evidence on the cost analysis of corneal tissue processing for transplantation?

- P (Population): Corneas donated for transplantation purposes.
- C (Concept): Cost analysis.
- C (Context): Processing of corneal tissue.

### Inclusion criteria

Full-text articles, company documents, manuals, guidelines, protocols, government documents, theses and dissertations available in full will be included, as long as they address the cost analysis of corneal tissue preparation processes for transplantation performed in eye banks, without restrictions on publication date or language. In addition, the reference lists of the publications will be reviewed to identify all studies related to the topic of interest.

The inclusion criteria according to the PCC mnemonic are as follows:

**Population:** Corneas harvested for transplantation purposes obtained from human donors.
**Concept:** cost analysis. Cost analysis consists in identifying, quantifying, and valuing all resources used in a given activity. It includes the assessment of physical resources (structure and equipment), human resources (professionals involved in the process), and consumables (supplies) [13].
**Context:** The processing of donated corneal tissue for transplantation involves activities performed in an eye bank to collect and prepare the cornea for the transplant procedure. This process follows rigorous steps which require adequate infrastructure, human resources, equipment, and supplies to ensure the quality and safety of the tissue that will be used for keratoplasty [14].

### Exclusion criteria

News articles, monographs, reviews, editorials, letters to the editor, expert opinions, abstracts, conference proceedings and correspondence will be excluded. Furthermore, as studies will be sourced from multiple databases, duplicates will also be excluded and studies considered only once.

### Information sources

The proxy of the Federal University of Mato Grosso do Sul (UFMS) will be used through the Journal Portal of the Coordination for the Improvement of Higher Education Personnel (CAPES) to systematically access the most comprehensive literature repositories in health sciences in order to obtain the information sources. CAPES maintains contracts with the most relevant scientific document repositories. Use of the CAPES journal portal provides users with access to paid and restricted content of the databases.

The following indexed literature databases will be used to apply the search strategy: MEDLINE/PubMed (via National Library of Medicine); SCOPUS (Elsevier); Science Direct (Elsevier); Web of Science Core Collection (Clarivate Analytics); Embase (Elsevier); Cochrane Library; Jama Network; Cell Press Collection; Ovid; Slack Inc; Cinahl.

Since the aim of a scoping review is to conduct the broadest possible search, the method also includes the use of unpublished primary sources of evidence, such as grey literature or hard-to-find documents. Therefore, searches will be conducted in the following repositories and databases: Grey Literature Information System in Europe; CAPES Catalogue of Theses and Dissertations; Open Access Scientific Repositories of Portugal (RCAAP); Institutional Repository of Theses and Research Papers of the National University Mayor of San Marcos (Cybertesis); National Library of Australia (Trove); Theses held in European repositories (DART EUROPE); Online Academic Archive (DIVA); NICE—National Institute for Excellence in Health and Care; NHS Economic Evaluation Database; Health Economic Evaluations; and the Canadian Agency for Drugs and Technologies in Health; Open Access Theses and Dissertations (OATD); World Health Organization (WHO); Eye Bank Association of America (EBAA); European Eye Bank Association (EEBA); Association for Research in Vision and Ophthalmology (ARVO); Centers for Disease Control and Prevention (CDC); and the National Eye Institute (NEI).

The manual search will be performed in the reference list of all documents included in the selection process to check for possible selection biases in the databases.

### Search strategy

A preliminary search was performed in the MEDLINE/PubMed (via the National Library of Medicine) and Web of Science databases to formulate the search strategy and verify controlled descriptors or keywords that could improve the strategy. A pilot search strategy was performed following the recommendations of the JBI Evidence Synthesis Manual in the MEDLINE/PubMed database (via the National Library of Medicine) on February 22, 2024, using the MeSH Controlled Descriptors to validate the strategy (see S2 Appendix). Searches in other databases mentioned in the “information sources” topic will only be performed after peer validation of this protocol.

### Data management

Mendeley^®^ Software will be used to remove duplicates. Rayyan^®^ Software will be used to analyze titles and abstracts blinded by two researchers. Data obtained from selected evidence sources will be tabulated using Microsoft Excel^®^.

### Screening process

The studies will be selected by two independent researchers who will work blindly. In the event of disagreement without consensus between them, a third reviewer (the study supervisor) will be responsible for the evaluation.

Files in databases where export tools for studies are available will be extracted individually and attached to the Mendeley^®^ Software for inclusion in the sample. The selection process will occur in three stages, as described below:

1. **Screening:** Retrieved documents will be selected using appropriate search strategies in the databases, based on the thresholds defined by the inclusion criteria.
2. **Eligibility:** Duplicates will be removed. The titles and abstracts of all documents retrieved from the databases will be read to assess their potential for inclusion in the review.
3. **Full-text reading:** After initial screening based on titles and abstracts, the selected full-texts will be read to confirm their relevance to the review.

The collected data will then be transcribed into a Microsoft Excel^®^ spreadsheet. The following information will initially be collected: Citation (authors); Year of publication; Origin (journal, guideline, thesis, among others); Location (country or region); The study objective; Study design; Population, Concept (type of cost analysis and values found), Context; Results and Limitations.

### Data synthesis

The PRISMA flowchart with extension for scoping reviews will be presented in order to detail the screening, eligibility and inclusion criteria. Next, the variables associated with the cost analyses of corneal tissue processing for transplants will be described. Finally, in the planned approach to presenting the data synthesis, descriptive tables, graphs and figures will be made that describe the measurement instruments used. There is no intention to perform a meta-analysis or synthesis of quantitative data.

### Expected results

This protocol is expected to provide a systematic framework for conducting a scoping review that provides an overview of the costs associated with corneal tissue processing and identifies gaps to support future cost analysis studies.

Potential limitations include the inherent characteristics of scoping reviews. There may also be considerable heterogeneity among the included studies due to their broad search criteria, with no restrictions on study designs and methods, which may affect interpretation of the results.

## Conclusion

With ever-increasing demand, healthcare systems are under pressure to establish efficient resource management. Shedding light on the costs of processing corneas for transplantation is an important step in filling gaps in knowledge and providing a basis for more assertive decisions. It is in this context that this scoping review aims to contribute with a synthesis of the global scientific production on the costs related to processing corneal tissues for transplantation as a guide for the scarce investment in healthcare.

## Supporting information

S1 Appendix Preferred Reporting Items for Systematic Reviews and Meta-Analyses Extension to Scoping Reviews (PRISMA-ScR) checklist. (DOCX)

S2 Appendix Pilot search strategy for the MEDLINE/PubMed database (via the National Library of Medicine). (DOCX)

S3 Appendix PRISMA-P (Preferred Reporting Items for Systematic Review and Meta-Analyses Protocols) checklist 2015: recommended items to address in a systematic review protocol. (DOCX)
