# Supplementary material for: Cost analysis of corneal tissue processing: A scoping review protocol
Source: PLoS One. 2025 Feb 10;20(2):e0317681. doi: 10.1371/journal.pone.0317681 (PMC11809855; doi:10.1371/journal.pone.0317681)
Supplement: S2 Appendix — (DOCX) [file pone.0317681.s002.docx]

**S2 Appendix**

Pilot search strategy for MEDLINE/PubMed database (via National Library of Medicine) on february 22, 2024.

"cornea"[MeSH Terms] AND ("Corneal Transplantation"[All Fields] OR "transplantation cornea"[All Fields] OR "Keratoplasty"[All Fields] OR "Cornea Transplantation"[All Fields] OR "Keratoplasties"[All Fields] OR "Cornea Transplantations"[All Fields] OR ("Corneal Transplantation"[MeSH Terms] OR ("corneal"[All Fields] AND "transplantation"[All Fields]) OR "Corneal Transplantation"[All Fields] OR ("transplantations"[All Fields] AND "cornea"[All Fields])) OR "transplantation corneal"[All Fields] OR "Corneal Transplantations"[All Fields] OR "transplantations corneal"[All Fields] OR "grafting corneal"[All Fields] OR "Corneal Grafting"[All Fields] OR ("Corneal Transplantation"[MeSH Terms] OR ("corneal"[All Fields] AND "transplantation"[All Fields]) OR "Corneal Transplantation"[All Fields] OR ("corneal"[All Fields] AND "graftings"[All Fields])) OR ("Corneal Transplantation"[MeSH Terms] OR ("corneal"[All Fields] AND "transplantation"[All Fields]) OR "Corneal Transplantation"[All Fields] OR ("graftings"[All Fields] AND "corneal"[All Fields])) OR "keratoplasty lamellar"[All Fields] OR ("Corneal Transplantation"[MeSH Terms] OR ("corneal"[All Fields] AND "transplantation"[All Fields]) OR "Corneal Transplantation"[All Fields] OR ("Keratoplasties"[All Fields] AND "lamellar"[All Fields])) OR "Lamellar Keratoplasties"[All Fields] OR "Lamellar Keratoplasty"[All Fields] OR "Eye Banks"[All Fields] OR "bank eye"[All Fields] OR "banks eye"[All Fields]) AND ("Costs and Cost Analysis"[All Fields] OR "costs cost analysis"[All Fields] OR "cost cost analysis"[All Fields] OR "Costs and Cost Analyses"[All Fields] OR "Cost Analysis"[All Fields] OR "analysis cost"[All Fields] OR "analyses cost"[All Fields] OR "Cost Analyses"[All Fields] OR "Cost Comparison"[All Fields] OR "comparison cost"[All Fields] OR "comparisons cost"[All Fields] OR "Cost Comparisons"[All Fields] OR "Affordability"[All Fields] OR ("Costs and Cost Analysis"[MeSH Terms] OR ("Costs"[All Fields] AND "Cost"[All Fields] AND "analysis"[All Fields]) OR "Costs and Cost Analysis"[All Fields]) OR "cost minimization analysis"[All Fields] OR "analyses cost minimization"[All Fields] OR "analysis cost minimization"[All Fields] OR "cost minimization analysis"[All Fields] OR "Cost-Minimization Analyses"[All Fields] OR "Pricing"[All Fields] OR "Cost"[All Fields] OR "Costs"[All Fields] OR "Cost Measures"[All Fields] OR "Cost Measure"[All Fields] OR "measure cost"[All Fields])

**130 documents recovered.**
